# Survey study of research integrity officers’ perceptions of research practices associated with instances of research misconduct

**DOI:** 10.1186/s41073-020-00103-1

**Published:** 2020-12-11

**Authors:** Michael Kalichman

**Affiliations:** Research Ethics Program, UC San Diego, 9500 Gilman Drive, La Jolla, CA 92093-0612 USA

**Keywords:** Good practices of research, Responsible conduct of research, Research integrity officer, Research misconduct

## Abstract

**Background:**

Research on research integrity has tended to focus on frequency of research misconduct and factors that might induce someone to commit research misconduct. A definitive answer to the first question has been elusive, but it remains clear that any research misconduct is too much. Answers to the second question are so diverse, it might be productive to ask a different question: What about how research is done allows research misconduct to occur?

**Methods:**

With that question in mind, research integrity officers (RIOs) of the 62 members of the American Association of Universities were invited to complete a brief survey about their most recent instance of a finding of research misconduct. Respondents were asked whether one or more good practices of research (e.g., openness and transparency, keeping good research records) were present in their case of research misconduct.

**Results:**

Twenty-four (24) of the respondents (39% response rate) indicated they had dealt with at least one finding of research misconduct and answered the survey questions. Over half of these RIOs reported that their case of research misconduct had occurred in an environment in which at least nine of the ten listed good practices of research were deficient.

**Conclusions:**

These results are not evidence for a causal effect of poor practices, but it is arguable that committing research misconduct would be more difficult if not impossible in research environments adhering to good practices of research.

## Introduction

Much of the literature on research misconduct has focused on the question of why a researcher might choose to engage in “fabrication, falsification, or plagiarism” (e.g., U.S. definition of research misconduct [1]). When cases of research misconduct reached the public eye in the 1980s, the scientific community saw such behavior as rare and likely the result of a few bad apples [2]. However, in a previous survey, as many as 1 in 3 researchers self-reported having engaged in serious misbehaviors, which included examples of research misconduct [3], perhaps an expected outcome of the research environment. Rates of research misconduct per se are probably much closer to 2% [4]. Even if just 2%, it would be hard to argue that these are simply a few damaged individuals. Whether the number is high or low, many have emphasized the importance of pressure in science for funding and publication (e.g., [5–7]). On the other hand, Fanelli et al. [8] concluded based on a retrospective meta-analysis of retractions that pressures to publish were less predictive of research misconduct than a variety of other environmental factors. One important factor might be the perceived fairness of the system [9–11].

Other studies of those who have been found to have committed research misconduct not surprisingly provide a more complicated picture in which possible factors resulting in misconduct are diverse [6, 12, 13]. Interestingly, many of the factors identified might be best classified as rationalizations (e.g., “I am a good person so the fact I did this must be because of some external factor such as I was forced to do so by others or the pressures of science”) or symptoms of less than ideal research environments (e.g., lack of mentoring). While these results are of interest, it is important to note that they are extracted from materials submitted in support of findings of research misconduct. Unfortunately, the primary charge to such reviews is typically only to determine if research misconduct occurred, not why it occurred. For example, institutions receiving funding from the Public Health Service are asked only to provide a “statement of findings”:

“*Statement of findings.* For each separate allegation of research misconduct identified during the investigation, provide a finding as to whether research misconduct did or did not occur … ” [14].

Despite the many possible factors that may have precipitated research misconduct, a response often cited as a solution is education (e.g., [15, 16]). In fact, institutions receiving funding from the Public Health Service are charged with fostering “a research environment that promotes the responsible conduct of research, research training, and activities related to that research or research training, discourages research misconduct …” [17]. However, if it is in fact just a few bad apples, or research misconduct is ubiquitous or a reaction to pressures, it isn’t readily apparent how education or training would be cost effective, much less useful at all (e.g., [18]). If education is a proposed solution, then presumably it is assumed that the problem is that scientists are insufficiently armed with knowledge or skills to protect them from committing research misconduct. While that is possible, it seems a tenuous conclusion given that the history of science prior to the 1980s was marked by the absence of such training.

It might be of use to reframe the question about research misconduct. Instead of asking “*Why* would a researcher commit misconduct?”, perhaps it would be of value to ask “*How is it possible* that a member of the scientific community *can* commit misconduct?” In short, shouldn’t the very nature of how research is done protect from the risk of misconduct? It may be worth considering the possibility that simply engaging in good practices of research would be a strong protection from research misconduct occurring – independent of the factors contributing to the misconduct. For example, if research is normally conducted in an environment in which researchers are open and transparent with one another, or if the responsibilities of authorship are emphasized at least as much as the credit, or if research records are kept sufficiently so that it is possible to reconstruct what had been done, then it seems any one of these good research practices would make it difficult if not impossible for a member of such a group to commit research misconduct.

The objective of this study is to supplement our understanding of the circumstances under which research misconduct typically occurs by obtaining perspectives of people overseeing research misconduct investigations about the characteristics of research practices in the environment in which research misconduct has occurred. It is hypothesized that research misconduct occurs in research environments that are characterized by deficient research practices.

## Methods

The components of this manuscript, including this methods section, address all applicable recommended items on the CHERRIES and STROBE checklists for reporting the results of Internet surveys [19] and cohort studies [20], respectively. This project was reviewed and approved for exempt status (45 CFR 46.101(b), category 2) by the UC San Diego Institutional Review Board (#170981XX).

A survey instrument was developed using SurveyMonkey for an audience of research integrity officers (RIOs), individuals with responsibility for receiving and responding to allegations of research misconduct. For the purpose of this manuscript, these individuals are referred to as “respondents,” a term which is sometimes used elsewhere to refer to an individual accused of having committed research misconduct. The complete survey is included as Additional file 1 (Research Misconduct Survey). The survey was designed to request perceptions of the extent to which research misconduct occurred in the context of good practices of research. Examples of good practices of research were selected based on the author’s observations made in teaching, publicly available information about research misconduct cases, and some personal experience with investigations into allegations of research misconduct. Draft survey questions were shared and finalized based on feedback from five leaders in the field of research integrity (named in the Acknowledgements). While the list of questions was not considered to be comprehensive or definitive, it was designed to address the range of issues that might be captured under the heading of “good practices of research.”

The survey was constructed so as to first verify that the respondent did have RIO responsibilities and had “firsthand knowledge of one or more cases of findings of research misconduct in [their] institution.” Adaptive (conditional) questioning was used only for the purpose of filtering out respondents who had not yet had responsibility for oversight of a research misconduct allegation. Those confirming were asked for their judgments regarding 13 factors possibly relevant to the most recent case of a research misconduct finding (Tables 1 and 2). The survey was delivered across six pages in SurveyMonkey and each survey page had at least one item and at most five items. With SurveyMonkey, respondents can correct or update answers by using the Back button. Each statement was scored for degree of agreement (Strongly disagree, Disagree, Neither agree nor disagree, Agree, Strongly Agree) or to be marked as something they didn’t know or was not applicable in the given case. The survey concluded with a request for their overall perception comparing “… the research practices [they] noted above to those in other research groups that have not experienced allegations or findings of research misconduct?” and an opportunity to submit an open-ended response to the prompt for “… additional comments or thoughts either about circumstances associated with research misconduct or this survey...” No personally identifiable information was collected and respondents were advised to not include any such information in open-ended responses.

**Table 1:** Good practices of research. Percent agreement that selected examples of good practices in research were present in the context of the case of research misconduct. Respondents were not included in the calculated percentages if they noted that they did not remember (or did not know about) a particular item or that it was not applicable

	Agree or Strongly Agree (n of N, %)	Don’t remember (n)	Not applicable (n)	No answer (n)
… open and transparent with each other about their work.	4 of 21, 19%	2	1	0
… had a good understanding of statistical methods or sought out the necessary expertise.	5 of 12, 42%	2	10	0
… considered authorship to be both a credit and a source of responsibility.	7 of 18, 39%	3	3	0
… felt empowered to speak up if something didn’t seem right or they had questions.	4 of 17, 24%	3	4	0
… leader of the research group/team was a good manager of:				
people.	5 of 21, 24%	0	1	2
budgets.	4 of 11, 36%	5	7	1
the research operations.	10 of 22, 45%	1	0	1
the research data.	6 of 21, 29%	1	1	1
… designed research studies to protect themselves from the risk of bias.	5 of 19, 26%	1	3	1
… kept research records sufficient for others to reconstruct what had or had not been done.	9 of 22, 41%	0	1	1

**Table 2:** Responsible conduct of research training. Percent agreement about whether the person found responsible for committing research misconduct had received one or more forms of responsible conduct of research (RCR) training. Respondents were not included in the calculated percentages if they noted that they did not remember (or did not know about) a particular item or that it was not applicable

	Agree or Strongly Agree (n of N, %)	Don’t remember (n)	Not applicable (n)	No answer (n)
… received adequate mentoring in the responsible conduct of research.	8 of 20, 40%	3	0	1
[took] one or more in person courses in responsible conduct of research.	9 of 14, 64%	9	0	1
[took] one or more online courses in responsible conduct of research (e.g., CITI).	6 of 12, 50%	11	0	1

Respondents to the survey were the RIOs associated with the 62 Association of American Universities (AAU) institutions. For each of these institutions, the RIO or equivalent was identified from the institution’s Web pages. This project was considered exempt for review by the Institutional Review Board, but a nominal informed consent process was included as part of the invitation (see Additional file 2 (E-mail Invitation).

A Research Integrity Officer or the equivalent (RIO) was identified for each of the 62 AAU institutions, but it was rarely easy to identify the right person from information on the Web. In many cases, it was necessary to take advantage of the author’s knowledge of the field and alternative key words.

On August 16, 2017, a single, personal e-mail invitation was sent to each of the 62 RIOs requesting their voluntary completion of the survey, open only to those invited. Unique logins were not required of respondents because the survey was short, the number of potential respondents was small, the survey was open for only a short period of time, and to help ensure anonymity. The survey was closed on September 16, 2017.

Responses were tabulated for reporting as percent of respondents agreeing or strongly agreeing with the statements listed.

## Results

Responses were received from 31 individuals (50% of those invited), all of whom self-identified as having responsibility or oversight for addressing allegations of research misconduct. Twenty-eight (28) of the respondents indicated they had dealt with at least one finding of research misconduct, but four of those did not answer the subsequent questions. All summaries below are based on the remaining 24 respondents (39% completion rate).

For those cases in which the RIOs rendered judgments, every one of the listed good practices of research were agreed to be present by less than half (19–45%) of the respondents (Table 1). The three categories of practices least frequently seen as characterizing the research environments for findings of research misconduct were that the researchers: (1) were open and transparent with each other about their work (4 of 21, 19% agreed or strongly agreed), (2) felt empowered to speak up if something didn’t seem right or they had questions (4 of 17, 24% agreed or strongly agreed), and (3) were in a group in which the group leader was a good manager of people (5 of 21, 24% agreed or strongly agreed) – all examples of people/social issues. The next two areas of greatest concern were both matters of data: (4) designing of research studies to protect from the risk of bias (5 of 19, 26% agreed or strongly agreed) and (5) effective management of the research data (6 of 21, 29% agreed or strongly agreed). Conversely, the statements with the highest rates of agreement (Table 2) were that researchers responsible for committing research misconduct had taken one or more in person courses in responsible conduct of research (9 of 14, 64% agreed or strongly agreed) or online courses in responsible conduct of research (6 of 12, 50% agreed or strongly agreed).

Over 25% of respondents judged that their case of research misconduct occurred in a context devoid of any of the ten good practices of research listed in Table 1. An additional 26% of respondents concluded that their case was characterized by only one of the ten good practices. Over 90% of the respondents concluded that the context for their research misconduct case was characterized by only five of the ten possible practices. Overall, most of the responding RIOs rated research practices in the environments in which they had a finding of research misconduct to be worse or much worse than average (14 of 21, 67%, worse or much worse), and very few (2 of 21, 10%, better or much better) rated the research practices to be better.

With 24 respondents and ten categories of research practices to be rated, this yielded a possible 240 answers. With 7 instances of no answer, and 31 not applicable, this provides a denominator of 202 questions answered. Out of these 202 questions, 18 responses were “don’t remember,” which means 8% of the time these were factors not clearly known/remembered by the RIOs.

The survey concluded with an open-ended question (“If you have additional comments or thoughts either about circumstances associated with research misconduct or this survey, please enter them here.”). Only seven of the 24 respondents referenced above answered this question. For completeness, all responses are included in Additional file 3 (Open-ended Responses). One of these responses clearly referenced the risk to research integrity when management and oversight are deficient:

“The PI managed a large group of researchers, but did not adequately manage or oversee their work to the extent where they were able to identify [questionable] research practices, nor were they able to produce proper research records when requested. Although it was misconduct, it stemmed mostly from careless management, mentoring, and research practices rather than an outright effort to deceive.”

Three of these responses referenced factors that to some degree could be a mismatch given the goals of this study:

“The most recent case involved plagiarism, but I realized that it didn’t fit well with your questions, so I switched to an earlier case.”

“The [accused] in this case had no moral compass. They created a story that they felt would play well with the committee members, colleagues and superiors out of whole fabric. It was only when this pathology became apparent, that the members of the committee and panel could step outside of norms of responsible conduct [to recognize] someone who did not respect those norms.”

“Because this survey was based on my most recent research misconduct case, I am afraid it [is] not very accurate. That case was a confession by someone who systematically changed data, sometimes for no apparent reason.”

## Discussion

Consistent with the hypothesized association, 28 RIO respondents perceived that their most recent finding of research misconduct occurred in circumstances in which good practices of research were absent. The premise of this study came from an insight from teaching responsible conduct of research that many recommended practices (e.g., being open and transparent, understanding and appropriate use of statistics, or keeping good records) would in theory make it harder for someone to commit research misconduct. In fact, it is easy to imagine that any one of the listed practices (Table 1) might be a serious impediment to such misconduct. While the present study was not a prospectively designed double blind trial – a study that would be difficult if not impossible to conduct – it does provide some insight into the circumstances present in a few recent cases of research misconduct in some of the leading academic universities of North America.

As summarized above, the environment in which these cases of research misconduct occurred was characterized by deficits in one or more of the selected practices noted in Table 1. In only one of the cases did the respondent note that all of these research practices were in place. Otherwise, more than half of the respondents were able to identify only one of the ten listed good practices as characterizing the circumstances in which the case of research misconduct had occurred. It is noteworthy that these items for the most part are not extraordinary expectations, but rather approaches to research that one might assume would be typical. Fortunately, only about 10% of the respondents believed that research practices surrounding their most recent research misconduct case were better than typical in the research environment.

While the list of good practices of research listed above is by no means comprehensive, it is perhaps not surprising that the practices most frequently seen as deficient could all be categorized as people/social issues: being open and transparent, feeling empowered to speak up if needed, and having a group in which the leader is a good manager of people. The next two practices most frequently seen as deficient were very clearly related to data: designing of research studies to protect from bias and effective data management. In all five of these cases, it is easy to see how much harder it would be for research misconduct to have occurred if these practices had been in place.

While protecting from the risk of research misconduct is an important goal, it is not the only possible benefit of improving standard practices of research. As noted in the open-ended response from one of the respondents, poor management and oversight can create an environment in which there is an increased risk of questionable research practices:

“The PI … Although it was misconduct, it stemmed mostly from careless management, mentoring, and research practices rather than an outright effort to deceive.”

In addition to the intended primary focus on good practices of research as a protection from research misconduct, four incidental observations are noteworthy. First, despite the possible relevance of these good practices to the risk of research misconduct, 8% of the possible answers about these good practices were marked as “Don’t know or don’t remember”. It seems as a minimum that such information would be an opportunity to better understand the circumstances that allowed research misconduct to occur. More importantly, this could be a window allowing for better design of options to strengthen the research enterprise and to protect against future cases. Perhaps questions about research practices should more routinely be part of all research misconduct investigations.

Second, related to the first finding, it is remarkable that at least half of the respondents did not provide ratings for three of the items they were asked about. That said, two of these three (allocation of funds and statistics) are not so surprising as ten and seven, respectively, of the RIOs concluded that the item was not applicable to their particular case. However, 9 of 23 respondents noted that they did not remember whether the individual in question had taken “one or more in person courses in responsible conduct of research.” It is of course possible that this item was simply forgotten even though it had been addressed at the time of the investigation. However, since so many of these RIOs readily reported on most of the other items listed, it seems more likely that the question of training in research ethics/integrity was simply not paramount in these research misconduct investigations. This is at the least surprising if it is assumed that training can help protect against the risk of research misconduct, which is the focus of the next finding.

A third incidental finding from this study is a reminder that we should not put too much stock in the protective value of training in responsible conduct of research. As summarized above, for those RIOs who responded, nearly two thirds of the individuals who had committed research misconduct had taken an in person course in responsible conduct of research and half had taken an online course in responsible conduct of research. Admittedly, the quality of courses may be a factor [21], but this finding is consistent with other studies that have emphasized the importance of what happens in the research environment rather than courses per se [22]. Nonetheless, it is a question worth investigating whether some forms of training might do better than others in mitigating the risk of research misconduct.

A fourth and final incidental finding from this project is that many institutions likely have room to improve in transparency about where and how to report allegations of research misconduct. Despite the author’s familiarity with the field, it was often challenging to find the right individual. Specific examples were not recorded at the time the study was conducted, but as recently as June 2020 Google searches for “Research Integrity Officer” at the 62 AAU institutions resulted in nine instances (=15%) with no hits. Unfortunately, even those with pages including the words “Research Integrity Officer” provided information about roles and/or responsibilities for the position but failed to clearly identify where the RIO could be found and how to lodge an allegation of research misconduct. To the extent this is true, it is another opportunity for improvement.

This study had at least four limitations that should be noted. First, it is far from comprehensive. Respondents represented only a sampling of North American universities. The survey asked only about the one, most recent case of research misconduct. The questions asked about good practices of research are only a sampling of the many practices that might be relevant to the responsible conduct of research. Nonetheless, the survey answers provided are sufficient to illustrate the principle that such factors are worth considering as being permissive if not supportive of cases of research misconduct.

Second, the focus of this survey was perceptions of these RIOs, not a definitive determination of whether these good practices of research were or were not present. Unfortunately, addressing the presence or absence of good practices of research is simply not paramount in investigations designed largely to assess whether or not research misconduct has occurred. In the absence of definitive cataloging of that information, this study is offered as an initial attempt to assess whether the hypothesis that the absence of such practices is permissive for research misconduct is worth pursuing based on the perceptions of these RIOs.

Third, one oversight of this study was the failure to distinguish between different forms of research misconduct. Although fabrication, falsification, and plagiarism are all considered research misconduct according to a government-wide definition in the US [1], plagiarism differs from the other two categories in that it does not necessarily involve a misrepresentation or manipulation of research data. To a large extent, fabrication and falsification were the intended focus for the present survey. Many of the questions clearly represent factors less likely to be relevant to plagiarism than to fabrication or falsification. It isn’t clear how many if any of the respondents were referencing plagiarism cases, but at least one noted the concern and because “it didn’t fit … switched to an earlier case.”

Fourth, although the premise of this study is to call attention to the possibility that the simple adoption of good practices of research would create an environment inhospitable to committing research misconduct, there are clearly cases in which that may be much less relevant. At least two such cases were cited in this study. The first is an instance in which the individual presumably recognized that they were violating accepted norms, but had no respect for them at all (“The [accused] in this case had no moral compass …” ). The second case was arguably someone who perhaps had no connection to questions of morality at all ( “… someone who systematically changed data, sometimes for no apparent reason.”).

Although both of the above cases might be instances in which the individual would not have been swayable by the good practices of their colleagues, it does still seem plausible that (for example) a collaborative and transparent research environment would have made it harder for them to commit their misconduct.

## Conclusion

While this study was not designed to prospectively test the association between research practices and cases of research misconduct, the perceptions of these RIO respondents are consistent with the hypothesis that research misconduct tends to occur in an environment in which research practices fall short. These results are not evidence for a causal effect of poor practices, but it is noteworthy that good research practices such as those listed would make it more difficult if not impossible for someone in such an environment to commit research misconduct. This perspective aligns with the recognition that an important factor in crime is having the opportunity [23]. This way of thinking about prevention of research misconduct almost certainly would not eliminate the possibility of research misconduct, but it would have several noteworthy features. First, fostering a research environment in which most members of the community adhere to good practices of research might fail to dissuade any one individual from committing research misconduct, but such an environment would make it more difficult for them to do so with impunity. Second, an emphasis on good research practices would create an environment that would mitigate against the risk of an ethical slippery slope [24]. Third, this approach is independent of the reasons for which someone might commit research misconduct. Good research practices would prevent, highlight, and/or mitigate the act of misconduct regardless of the reason it was committed. Finally, whether or not research misconduct would be diminished by increased emphasis on good practices of research, it seems clear that the integrity and reproducibility of science could only be helped by improving the way in which we do science. Given the current increased focus on research rigor and reproducibility (e.g., [25]), it is worth asking if we can better design programs (including RCR training) that will foster a climate in which good practices of research will be more widely adopted, thereby decreasing the risk of research misconduct, but at the same time increasing the likelihood of high quality research.

## Supplementary Information

**Additional file 1.** Research Misconduct Survey.

**Additional file 2.** E-mail Invitation.

**Additional file 3.** Open-ended Responses.

## Data Availability

The survey and all anonymized survey responses are available on request.

## References

[1] Office of Science and Technology Policy (2000). Federal policy on research misconduct. Fed Regist.

[2] Broad WJ (1981). Congress told fraud issue ‘exaggerated’. Science.

[3] Martinson BC, Anderson MS, De Vries R (2005). Scientists behaving badly. Nature.

[4] Fanelli D (2009). How many scientists fabricate and falsify research? A systematic review and meta-analysis of survey data. PLoS One.

[5] Mumford MD, Connelly MS, Helton WB, Strange JM, Osburn HK (2001). On the construct validity of integrity tests: individual and situational factors as predictors of test performance. Int J Sel Assess.

[6] Davis MS, Riske-Morris M, Diaz SR (2007). Causal factors implicated in research misconduct: evidence from ORI case files. Sci Eng Ethics.

[7] Tijdink JK, Verbeke R, Smulders YM (2014). Publication pressure and scientific misconduct in medical scientists. J Empir Res Hum Res Ethics.

[8] Fanelli D, Costas R, Larivière V (2015). Misconduct policies, academic culture and career stage, not gender or pressures to publish, affect scientific integrity. PLoS One.

[9] Keith-Spiegel P, Koocher GP (2005). The IRB paradox: could the protectors also encourage deceit?. Ethics Behav.

[10] Martinson BC, Anderson MS, Crain AL, De Vries R (2006). Scientists’ perceptions of organizational justice and self-reported misbehaviors. J Empir Res Hum Res Ethics.

[11] Martinson BC, Crain AL, De Vries R, Anderson MS (2010). The importance of organizational justice in ensuring research integrity. J Empir Res Hum Res Ethics.

[12] Wright DE, Titus SL, Cornelison JB (2008). Mentoring and research misconduct: an analysis of research mentoring in closed ORI cases. Sci Eng Ethics.

[13] DuBois JM, Anderson EE, Chibnall J, Carroll K, Gibb T, Ogbuka C, Rubbelke T (2013). Understanding research misconduct: a comparative analysis of 120 cases of professional wrongdoing. Account Res.

[14] Code of Federal Regulations. Institutional investigation report. The institutional investigation § 93.313(f); 2011. p. 660. https://www.govinfo.gov/content/pkg/CFR-2011-title42-vol1/pdf/CFR-2011-title42-vol1-sec93-314.pdf.

[15] Resnik DB, Master ZM. Policies and initiatives aimed at addressing research misconduct in high-income countries. PLoS Med. 2013. 10.1371/journal.pmed.1001406. 10.1371/journal.pmed.1001406 PMC3608541 23555198

[16] Yeo-Teh NSL, Tang BL. Research ethics courses as a vaccination against a toxic research environment or culture: Sage Publications; 2020. p. 1–11. 10.1177/1747016120926686. © The Authors 2020.

[17] Code of Federal Regulations. Compliance and assurances. Subpart C- responsibilities of institutions. § 93.300(c); 2011. p. 655. https://www.govinfo.gov/content/pkg/CFR-2011-title42-vol1/pdf/CFR-2011-title42-vol1-sec93-300.pdf.

[18] Marusic A, Wager E, Utrobicic A, Rothstein HR, Sambunjak D. Interventions to prevent misconduct and promote integrity in research and publication. Cochrane Database Syst Rev. 2016;(4). 10.1002/14651858.MR000038.pub2. 10.1002/14651858.MR000038.pub2 PMC7149854 27040721

[19] Eysenbach G (2004). Improving the quality of web surveys: the checklist for reporting results of internet e-surveys (CHERRIES). J Med Internet Res.

[20] von Elm E, Altman DG, Egger M, Pocock SJ, Gøtzsche PC, Vandenbroucke JP (2007). STROBE initiative. The strengthening the reporting of observational studies in epidemiology (STROBE) statement: guidelines for reporting observational studies. PLoS Med.

[21] Antes AL, Murphy ST, Waples EP, Mumford MD, Brown RP, Connelly S, Devenport LD (2009). A meta-analysis of ethics instruction effectiveness in the sciences. Ethics Behav.

[22] Anderson MS, Horn AS, Risbey KR, Ronning EA, De Vries R, Martinson BC (2007). What do mentoring and training in the responsible conduct of research have to do with scientists’ misbehavior? Findings from a national survey of NIH-funded scientists. Acad Med.

[23] Adams D, Pimple K (2005). Research misconduct and crime lessons from criminal science on preventing misconduct and promoting integrity. Account Res.

[24] Welsh DT, Ordóñez LD, Snyder DG, Christian MS (2015). The slippery slope: how small ethical transgressions pave the way for larger future transgressions. J Appl Psychol.

[25] Baker M (2016). Is there a reproducibility crisis?. Nature.

