# Supplementary material for: Survey study of research integrity officers’ perceptions of research practices associated with instances of research misconduct
Source: Res Integr Peer Rev. 2020 Dec 11;5:17. doi: 10.1186/s41073-020-00103-1 (PMC7731550; doi:10.1186/s41073-020-00103-1)
Supplement: Supplementary file 1 — Additional file 1. Research Misconduct Survey. [file 41073_2020_103_MOESM1_ESM.pdf]

Research Misconduct Survey

The purpose of this survey is to better understand *the context in which research misconduct occurs*.

Anonymity: **To avoid any risk of compromising your anonymity in completing this survey, no questions ask for identifiable personal or institutional demographic information, and your response to this survey cannot be tracked to you individually.**

Comments: **If you wish to qualify or comment on any or all of your answers, please note that there is an opportunity to do so with a comment box at the end of the survey.**

1. Do you have responsibility or oversight for conducting investigations of research misconduct at your institution?

- ◯ Yes
- ◯ No

2. In your role, do you have firsthand knowledge of one or more cases of findings of research misconduct in your institution?

- ◯ Yes
- ◯ No

Please answer the following questions ***based on what you know of the most recent finding of research misconduct in your institution***.

In your best judgment, to what extent was the context of this research misconduct finding characterized by each of the practices noted below.

All members of the research group or team:

3. were open and transparent with each other about their work.

- Strongly Disagree
- Disagree
- Neither Agree nor Disagree
- Agree
- Strongly Agree
- Don't remember or don't know
- Not applicable

4. either had a good understanding of statistical methods or sought out the necessary expertise.

- Strongly Disagree
- Disagree
- Neither Agree nor Disagree
- Agree
- Strongly Agree
- Don't remember or don't know
- Not applicable

5. considered authorship to be both a credit and a source of responsibility.

- Strongly Disagree
- Disagree
- Neither Agree nor Disagree
- Agree
- Strongly Agree
- Don't remember or don't know
- Not applicable

6. felt empowered to speak up if something didn’t seem right or they had questions.

- Strongly Disagree
- Disagree
- Neither Agree nor Disagree
- Agree
- Strongly Agree
- Don't remember or don't know
- Not applicable

Research Misconduct Survey

Please answer the following questions ***based on what you know of the most recent finding of research misconduct in your institution***.

In your best judgment, to what extent was the context of this research misconduct finding characterized by each of the practices noted below.

The leader of the research group/team was a good manager of:

7. people.

- Strongly Disagree
- Disagree
- Neither Agree nor Disagree
- Agree
- Strongly Agree
- Don't remember or don't know
- Not applicable

8. budgets.

- Strongly Disagree
- Disagree
- Neither Agree nor Disagree
- Agree
- Strongly Agree
- Don't remember or don't know
- Not applicable

9. the research operations.

- Strongly Disagree
- Disagree
- Neither Agree nor Disagree
- Agree
- Strongly Agree
- Don't remember or don't know
- Not applicable

10. the research data.

- Strongly Disagree
- Disagree
- Neither Agree nor Disagree
- Agree
- Strongly Agree
- Don't remember or don't know
- Not applicable

Research Misconduct Survey

Please answer the following questions based on what you know of the most recent finding of research misconduct in your institution.

In your best judgment, to what extent was the context of this research misconduct finding characterized by each of the practices noted below.

The respondent(s) to this research misconduct allegation:

11. designed research studies to protect themselves from the risk of bias.

- Strongly Disagree
- Disagree
- Neither Agree nor Disagree
- Agree
- Strongly Agree
- Don't remember or don't know
- Not applicable

12. kept research records sufficient for others to reconstruct what had or had not been done.

- Strongly Disagree
- Disagree
- Neither Agree nor Disagree
- Agree
- Strongly Agree
- Don't remember or don't know
- Not applicable

13. had received adequate mentoring in the responsible conduct of research.

- Strongly Disagree
- Disagree
- Neither Agree nor Disagree
- Agree
- Strongly Agree
- Don't remember or don't know
- Not applicable

14. had taken one or more in person courses in responsible conduct of research.

- Strongly Disagree
- Disagree
- Neither Agree nor Disagree
- Agree
- Strongly Agree
- Don't remember or don't know
- Not applicable

15. had taken one or more online courses in responsible conduct of research (e.g., CITI).

- ◯ Strongly Disagree
- ◯ Disagree
- ◯ Neither Agree nor Disagree
- ◯ Agree
- ◯ Strongly Agree
- ◯ Don't remember or don't know
- ◯ Not applicable

Research Misconduct Survey

16. In your judgment and experience, how would you compare the research practices you noted above to those in other research groups that have not experienced allegations or findings of research misconduct?

- ◯ Much worse
- ◯ Worse
- ◯ About the same
- ◯ Better
- ◯ Much better
- ◯ Don't remember or don't know
- ◯ Not applicable

17. If you have additional comments or thoughts either about circumstances associated with research misconduct or this survey, please enter them here.

Thank you very much for your time in completing this survey.

For further information, please contact Michael Kalichman <mkalichman@ucsd.edu>, director, UC San Diego Research Ethics Program
