# Supplementary material for: Survey study of research integrity officers’ perceptions of research practices associated with instances of research misconduct
Source: Res Integr Peer Rev. 2020 Dec 11;5:17. doi: 10.1186/s41073-020-00103-1 (PMC7731550; doi:10.1186/s41073-020-00103-1)
Supplement: Supplementary file 2 — Additional file 2. E-mail Invitation. [file 41073_2020_103_MOESM2_ESM.pdf]

E-mail Invitation

The following is the complete text of the e-mail sent to Research Integrity Officers to invite their participation in this survey study:

> I am writing to ask your help as a representative of [your institution], one of the 62 members of the Association of American Universities.
> You are being invited to participate in a research study titled “Reported perspectives on circumstances associated with cases of research misconduct” that is being conducted to better understand the circumstances under which research misconduct occurs. Based on publicly available information on your institution’s website, I identified you as the institutional official likely serving as the Research Integrity Officer (RIO), with responsibility for oversight of allegations of research misconduct.
> As RIO, you are being contacted to see if you would be interested in participating in this study. If you are not the RIO, could you please refer me to the correct individual?
> I am conducting this study to offer feedback on the results as part of this September’s meeting of the Association for Research Integrity Officers, as well as possible publication. It will require that you complete a brief survey at:
> https://www.surveymonkey.com/r/research_misconduct
> This study was reviewed and certified as exempt from IRB review under 45 CFR 46.101(b), category 2 by the UC San Diego Human Research Protections Program. The study is being done by Michael Kalichman, PhD, director of the UC San Diego Research Ethics Program
> Research records will be kept confidential to the extent allowed by law and no one will know whether or not you participated in the study. There is little or no chance that your personal responses will be compromised, as your survey responses are not linked to you, minimal demographic information is collected, and survey results are retained only by the Principal Investigator for this project on secure password protected devices and servers.
> Participation in research is entirely voluntary. You may refuse to participate or withdraw at any time by simply exiting the survey. You are free to skip any question that you choose.
> If you have any questions about the study, you may reach Michael Kalichman at 858-822-2027. If you do not wish to participate, you do not need to do anything further. Please note that by completing and submitting the survey you are indicating that you are at least 18 years old and agree to participate in this research study.
