# Supplementary material for: Survey study of research integrity officers’ perceptions of research practices associated with instances of research misconduct
Source: Res Integr Peer Rev. 2020 Dec 11;5:17. doi: 10.1186/s41073-020-00103-1 (PMC7731550; doi:10.1186/s41073-020-00103-1)
Supplement: Supplementary file 3 — Additional file 3. Open-ended Responses. [file 41073_2020_103_MOESM3_ESM.pdf]

Open-ended Responses

The following is the complete text of comments made by 7 of the 24 respondents included in this study in response to the query:

If you have additional comments or thoughts either about circumstances associated with research misconduct or this survey, please enter them here.

1. Our investigation process does not delve into most of the issues raised above. Occasionally we learn that a laboratory has had budgetary constraints or that a particular person may have had concerns about speaking up, but we could only guess as to whether these issues created a climate that resulted in falsification, fabrication, or plagiarism. Also in the course of an investigation we do not routinely ask respondents if they have completed on-line or in-person RCR courses, and don't have systems in place that would permit us to easily acquire that information. Another thought: The ORI website has a list of recent findings of research misconduct with the names of the respondents. I understand that this is a skewed sample as it relates only to those cases over which PHS has jurisdiction, and not all of the respondents may be willing to participate in the survey. However, if you can reach them and if they respond, you may learn more from them than from RIOs and those who support them.

2. The PI managed a large group of researchers, but did not adequately manage or oversee their work to the extent where they were able to identify [questionable] research practices, nor were they able to produce proper research records when requested. Although it was misconduct, it stemmed mostly from careless management, mentoring, and research practices rather than an outright effort to deceive.

3. Q3. Certainly one member of the team was not open and transparent. That person was found to have committed research misconduct. Q6. Yes, some were comfortable speaking up. Q12. The questions seem to be framed for a case where the respondent was the head of the lab.

4. The most recent case involved plagiarism, but I realized that it didn't fit well with your questions, so I switched to an earlier case. But this might confound your results--probably too late, but might have been helpful to first ask what the primary allegations involved (F, F or P).

5. Case involved a new investigator who had intense pressures to succeed

6. Because this survey was based on my most recent research misconduct case, I am afraid it not very accurate. That case was a confession by someone who systematically changed data, sometimes for no apparent reason. Looking at all of the many cases I handle, most stem from [inadequate] supervision of lab personnel and failure to monitor raw data and compare that raw data to data presented at lab meetings, etc. In my most recent case, analysis of raw data also would have caught the misconduct much sooner.

7. The respondent in this case had no moral compass. They created a story that they felt would play well with the committee members, colleagues and superiors out of whole fabric. It was only when this pathology became apparent, that the members of the committee and panel could step outside of norms of responsible conduct by someone who did not respect those norms.
